# Supplementary material for: Spillover effects of violent attacks and COVID-19 exposure on mental health of health professionals: A two-phase quasi-natural experiments study in Northwest China
Source: Glob Ment Health (Camb). 2023 Oct 26;10:e76. doi: 10.1017/gmh.2023.65 (PMC10685256; doi:10.1017/gmh.2023.65)
Supplement: Liu et al. supplementary material [file S2054425123000651sup001.docx]

# Appendix A: Model specifications

*Study I*. In study I, we only estimate the impact of MV on the MH of HPs using 2019 data. Because the variable denoting the intensity of MV is the regional number of lawsuit cases, we hypothesize that every HP in TS was equivalently exposed to risks from MV and estimate the following regression:

$mhealth_{ij}=\alpha+\sum_{\gamma=1}^{3} \beta_{\gamma}Cases^{ts}\times Role_{ij}^{\gamma}+X_{ij}^{'}\Gamma+\delta_{j}+\varepsilon_{ij}$. (1)

where $mhealth_{ij}$ is the outcome variable MH for HP $i$ at hospital $j$. $Cases^{ts}$ is the number of lawsuit cases about MV in TS, and $Role_{ij}^{\gamma}$ indicates the role of HP $i$ at hospital $j$. To distinguish the risk of exposure to MV, we categorized HPs into three groups: physicians, nurses, and others denoted by $\gamma\in\{1,2,3\}$. We define physicians and nurses as frontier HPs; the last group is the reference.

$X_{ij}^{'}$ is a set of individual-level covariates, including age, gender, education level, income, marital status, and professional rank (*Zhicheng* in Chinese), following workplace violence risk factors in the healthcare setting ^15^. $\alpha$ and $\varepsilon_{ij}$ indicate the constant term and the error term, respectively. $\delta_{j}$is the hospital fixed effects absorbing potential unobservable differences among hospitals, which can affect HP’s MH. $\beta_{\gamma}$ is the coefficient of interest capturing the extent of the impact of MV on physicians and nurses relative to other non-frontier HPs.

*Study II*. Study II allows us to explore the regional variation in MV and COVID-19 exposure on HPs’ MH with 2020 survey data. We begin our estimation by the following equations (2) and (3) to separately examine the potential impact of MV and COVID-19 in 2020.

$mhealth_{ij}=\alpha+\beta Cases_{j}+X_{ij}^{'}\Gamma+\delta_{j}+\varepsilon_{ij}$ (2)

$mhealth_{ij}=\alpha+\beta Covid_{j}+X_{ij}^{'}\Gamma+\delta_{j}+\varepsilon_{ij}$ (3)

Here, $mhealth_{ij}$ is the outcome variable MH for HP $i$ in prefecture city $j$. $Cases_{j}$ and $Covid_{j}$ are the number of lawsuit cases about MV and the confirmed COVID-19 cases in city $j$, respectively. $X_{ij}^{'}$ is also a set of individual-level covariates, including age, gender, education level, income, marital status, and professional rank (*Zhicheng* in Chinese).

$\alpha$ and $\varepsilon_{ij}$ indicate the constant term and the error term. $\delta_{j}$ are regional fixed effects absorbing potential unobservable differences among cities, which can affect HPs’ MH. $\beta$ is the coefficient of interest capturing the extent of the impact of MV or COVID-19 on HPs. Because of the concern that some HPs may hesitate to report the hospitals they belonged to, disclosing hospitals was optional in this round survey. Therefore, the hospital-fixed effects were no longer controlled due to much missing information.

Media reports of anecdotes show that MV did not cease during the pandemic, so another possible concern is the interaction effects of MV and COVID-19. To test whether COVID-19 has exacerbated the impact of MV on HP health, we estimate the following Equation (4):

$mhealth_{ij}=\alpha+\beta Covid_{j}\times Cases_{j}+X_{ij}^{'}\Gamma+\delta_{j}+\varepsilon_{ij}$ (4)

where $Covid_{j}\times Cases_{j}$ is the interaction between the number of lawsuit cases about MV and the number of confirmed COVID-19 cases in city $j$ in 2020. The coefficient $\beta$, as above, represents how MV and COVID-19 jointly affected HP’s health.

Beyond the above analysis, the 2020 survey provides an opportunity for the same estimation as shown in Equation (1). The effects of MV, COVID-19, and their interaction on the MH of different HP groups can be examined. HPs were categorized into three groups (physicians, nurses, and others) by whether they were exposed to the frontier of pandemic risks. Similarly, we assume that physicians take the highest risk, nurses follow, and the non-frontier HPs are base-reference. Remarkably, the regional variation of treatment variables imposes no identical impact across the sample other than Study I as an essential supplement.

*Study III*. Although two of our surveys are cross-sectional, the before and after COVID-19 set allows us to compare this pandemic’s potential effects on HPs’ MH with the 2019 and 2020 data as additional evidence. Following the strategy of Azoulay, Stuart ^16^, we also use coarsened exact matching (CEM) to match the 2019 and 2020 data. CEM, a nonparametric matching approach, can conserve sample size with less dependence on regression models and is easier to reach the principle of consistency than regular match approaches such as propensity score matching ^17,18^. We mainly matched the 2019 data in TS with the 2020 data in LZ because they are the two largest cities in GS and have approximate levels of hospital distribution.

Because no variation in lawsuit cases of MV for TS and LZ can be found in 2019 and 2020 (although regional variation remained), Study III mainly estimates the effects of COVID-19. Like Azoulay, Stuart ^16^, we use Equation (5) to estimate the HP-pair level.

$E[\triangle mhealth_{i(i)j}|X_{ij}]=\alpha+\beta Covid_{j}+X_{ij}^{'}\Gamma+\delta_{j}+\varepsilon_{ij}$ (5)

The outcome viable $E[\triangle mhealth_{i(i)j}|X_{ij}]$ ($\triangle mhealth_{i(i)j}=mhealth_{ij}-mhealth_{(i)j}$) indicates the difference in MH indicators between a treated HP $i$ and the corresponding control HP $(i)$ matched by CEM. $Covid_{j}$ here denote the treatment effects of COVID-19 and switching to 1 for HPs from LZ. The interaction of COVID-19 with cases (from regional variation) and the role of HPs were also analyzed.

# Appendix B: Tables and figures

Table A1 Description of SRHMS (mental health)

| Indicators | Items | Aim |
| --- | --- | --- |
| Positive  emotion | 1. Are you optimistic about the future? 2. Are you satisfied with your present living situation? 3. Are you confident about yourself? 4. Do you feel safe in your daily living environment? 5. Do you have a feeling of happiness? | Measuring positive mental conditions |
| Symptoms and negative  emotion | 1. Do you feel nervous? 2. Do you feel down in the dumps? 3. Are you afraid for no reason? 4. Do you have to be sure about what you have done? 5. Do you feel lonely when you are with others? 6. Do you feel restless and uneasy? 7. Do you feel empty and bored or have nothing to live for? | Measuring adverse mental conditions and psychological problems |
| Cognitive  function | 1. How is your memory? 2. Do you easily concentrate on one thing? 3. How well do you think or deal with problems? | Measuring the ability of cognition |
| Role activity  and social adaptability | 1. Can you properly deal with the unpleasant things that happen to you in your life, study, and work? 2. Can you quickly adapt to the new living, studying, and working environment? 3. How do you evaluate your role in work, life, and study? | Measuring the ability of role in activities and social adaptability |
| Social resource  and social  contact | 1. Is your family life harmonious? 2. Do you have many close colleagues, classmates, neighbors, relatives, or associates? 3. Do you have a friend who can share happiness and sadness with you? 4. Do you talk about problems with your friends or relatives? 5. Do you often keep in touch with your relatives and friends (such as visiting each other, calling, writing, etc.)? 6. Do you frequently participate in social and collective activities (such as caucuses, unions, student unions, religions, gatherings of friends, sports competitions, etc.)? | Measuring the ability to interact with family, friends, and others |
| Social support | 1. Can you rely on your family to a great extent when you need help? 2. Can you rely on your friends to a great extent when you need help? 3. When you are in trouble, do you take the initiative to seek help from others? 4. How do you feel about your general social functioning (interpersonal relationships, social interactions, etc.) compared with your peers? | Measuring the ability to provide support to family, friends, and others |

Table A2 Confirmed COVID-19 cases and cases of lawsuits in GS

| City | # Confirmed Covid-19 cases, 2020 | #Cases of lawsuit, 2019 | #Case of lawsuit, 2020 |
| --- | --- | --- | --- |
| Baiyin | 4 | 21 | 21 |
| Dingxi | 9 | 21 | 24 |
| Gannan | 8 | 2 | 1 |
| Jiayuguan | 0 | 2 | 5 |
| Jinchang | 1 | 4 | 6 |
| Jiuquan | 0 | 28 | 17 |
| Lanzhou | 127 | 76 | 76 |
| Longnan | 3 | 9 | 3 |
| Longxi | 4 | 15 | 6 |
| Pingliang | 9 | 28 | 18 |
| Qingyang | 3 | 22 | 23 |
| Tianshui | 12 | 22 | 19 |
| Wuwei | 0 | 15 | 10 |
| Zhangye | 2 | 4 | 9 |

Table A3 Results of Studies I and II

|  | Positive emotion | Negative emotion | Cognition | Social adaptability | Social contact | | Social  support |
| --- | --- | --- | --- | --- | --- | --- | --- |
|  | (1) | (2) | (3) | (4) | (5) | | (6) |
|  | Panel A: Study I | | | | | | |
| *Cases×physician* | -0·22*** | 0·23*** | -0·01 | -0·09*** | -0·08 | | -0·11* |
|  | (-0·26, -0·18) | (0·19, 0·28) | (-0·05, 0·04) | (-0·115, -0·055) | (-0·13, -0·03) | | (-0·18, -0·05) |
| *Cases×nurses* | -0·07* | 0·29*** | 0·02 | -0·03 | 0·04 | | -0·00 |
|  | (-0·11, -0·03) | (0·23, 0·35) | (-0·03, 0·06) | (-0·06, 0·00) | (-0·01, 0·08) | | (-0·06, 0·06) |
| Observations | 5,304 | 5,304 | 5,304 | 5,304 | 5,304 | | 5,304 |
| R-squared | 0·052 | 0·039 | 0·036 | 0·031 | 0·036 | | 0·038 |
| Mean of dependent variable | 7·230 | 5·280 | 6·440 | 7·510 | 6·950 | | 6·640 |
|  | Panel B: Study II-COVID-19 | | | | | | |
| *Covid-19* | 0·00 | 0·18*** | -0·03** | 0·06*** | -0·06*** | 0·06*** | |
|  | (-0·02, 0·03) | (0·16, 0·19) | (-0·05, -0·02) | (0·05, 0·08) | (-0·07, -0·05) | (0·05, 0·06) | |
| Observations | 3,883 | 3,883 | 3,883 | 3,883 | 3,883 | 3,883 | |
| R-squared | 0·030 | 0·019 | 0·044 | 0·024 | 0·025 | 0·016 | |
| Mean of dependent variable | 7·310 | 5·520 | 6·400 | 7·780 | 6·810 | 6·570 | |
|  | Panel C: Study II-MV | | | | | | |
| *Cases* | 0·01 | 0·20*** | -0·03** | 0·08*** | -0·07*** | 0·06*** | |
|  | (-0·01, 0·03) | (0·18, 0·21) | (-0·04, -0·02) | (0·07, 0·08) | (-0·07, -0·06) | (0·05, 0·07) | |
| Observations | 5,589 | 5,589 | 5,589 | 5,589 | 5,589 | 5,589 | |
| R-squared | 0·025 | 0·021 | 0·036 | 0·016 | 0·015 | 0·014 | |
| Mean of dependent variable | 7·320 | 5·590 | 6·470 | 7·760 | 6·790 | 6·590 | |
|  | Panel D: Study II-MV*×*COVID-19 | | | | | | |
| *Covid-19×cases* | 0·00 | 0·24*** | -0·04** | 0·08*** | -0·08*** | 0·07*** | |
|  | (-0·03, 0·03) | (0·22, 0·26) | (-0·06, -0·03) | (0·06, 0·10) | (-0·09, -0·07) | (0·07, 0·08) | |
| Observations | 3,851 | 3,851 | 3,851 | 3,851 | 3,851 | 3,851 | |
| R-squared | 0·028 | 0·019 | 0·043 | 0·022 | 0·022 | 0·015 | |
| Mean of dependent variable | 7·310 | 5·520 | 6·410 | 7·790 | 6·810 | 6·580 | |

Notes: 1. This table presents the results of point estimates shown in Figures 1 and 2. 2. *=p<0·1, **=p<0·05, ***=p<0·01. 3. The brackets under coefficients are the 95% Confidence Interval. 4. Standard errors are clustered at hospital levels in Study I and at city levels in Study II. 5. Cases=the lawsuit cases of MV, Covid-19=the number of confirmed regional Covid-19 cases.

Table A4 Results of Study III

|  | Positive emotion | Negative emotion | Cognition | Social adaptability | Social contact | Social  support |
| --- | --- | --- | --- | --- | --- | --- |
|  | (1) | (2) | (3) | (4) | (5) | (6) |
|  | Panel A | | | | | |
| *Covid-19* | -0·70* | 0·66** | -0·84*** | -0·39*** | -1·12*** | -0·89** |
|  | (-0·78, -0·63) | (0·64, 0·67) | (-0·85, -0·83) | (-0·40, -0·39) | (-1·13, -1·12) | (-0·91, -0·88) |
| Observations | 6,886 | 6,886 | 6,886 | 6,886 | 6,886 | 6,886 |
| R-squared | 0·009 | 0·008 | 0·017 | 0·013 | 0·007 | 0·004 |
|  | Panel B | | | | | |
| *Covid-19×cases* | -0·24* | 0·22** | -0·29*** | -0·13*** | -0·38*** | -0·30** |
|  | (-0·26, -0·21) | (0·22, 0·23) | (-0·29, -0·28) | (-0·14, -0·13) | (-0·38, -0·38) | (-0·31, -0·30) |
| Observations | 6,886 | 6,886 | 6,886 | 6,886 | 6,886 | 6,886 |
| R-squared | 0·009 | 0·008 | 0·017 | 0·013 | 0·007 | 0·004 |
|  | Panel C | | | | | |
| *Covid-19×physicians* | -0·86* | 0·67*** | -0·94*** | -0·50** | -1·18*** | -0·91** |
|  | (-0·95, -0·76) | (0·67, 0·68) | (-0·95, -0·94) | (-0·53, -0·48) | (-1·20, -1·17) | (-0·94, -0·88) |
| *Covid-19×nurses* | -0·46 | 0·92** | -0·71*** | -0·21* | -0·82*** | -0·53** |
|  | (-0·56, -0·36) | (0·90, 0·95) | (-0·71, -0·70) | (-0·23, -0·19) | (-0·83, -0·82) | (-0·55, -0·50) |
| *Covid-19×others* | -0·48** | 0·55* | -0·69** | -0·24* | -1·10** | -0·96*** |
|  | (-0·51, -0·46) | (0·48, 0·62) | (-0·73, -0·65) | (-0·26, -0·22) | (-1·12, -1·07) | (-0·97, -0·95) |
| Observations | 6,875 | 6,875 | 6,875 | 6,875 | 6,875 | 6,875 |
| R-squared | 0·013 | 0·009 | 0·018 | 0·016 | 0·009 | 0·008 |
|  | Panel D | | | | | |
| *Covid-19×cases×physicians* | -0·28* | 0·23** | -0·31*** | -0·16** | -0·40*** | -0·31** |
|  | (-0·30, -0·25) | (0·22, 0·23) | (-0·31, -0·31) | (-0·16, -0·15) | (-0·40, -0·39) | (-0·32, -0·30) |
| *Covid-19×cases×nurses* | -0·18 | 0·29*** | -0·25*** | -0·09** | -0·31*** | -0·22** |
|  | (-0·21, -0·15) | (0·28, 0·29) | (-0·26, -0·25) | (-0·10, -0·09) | (-0·32, -0·31) | (-0·23, -0·21) |
| *Covid-19×cases×others* | -0·19* | 0·20** | -0·25** | -0·10** | -0·38*** | -0·32** |
|  | (-0·20, -0·17) | (0·18, 0·21) | (-0·26, -0·24) | (-0·10, -0·09) | (-0·38, -0·37) | (-0·32, -0·31) |
| Observations | 6,875 | 6,875 | 6,875 | 6,875 | 6,875 | 6,875 |
| R-squared | 0·013 | 0·009 | 0·018 | 0·016 | 0·009 | 0·008 |

Notes: 1. This table presents the results of point estimates shown in Figure 3. 2. *=p<0·1, **=p<0·05, ***=p<0·01. 3. The brackets under coefficients are the 95% Confidence Interval. 4. Cases=the lawsuit cases of MV, Covid-19=the number of confirmed regional Covid-19 cases.

Table A5 Results of Robustness Check

|  | K10 | Positive emotion | Negative emotion | Cognition | Social adaptability | Social contact | Social  support |
| --- | --- | --- | --- | --- | --- | --- | --- |
|  | (1) | (2) | (3) | (4) | (5) | (6) | (7) |
| Panel A: Study I | | | | | | | |
| *Cases×physician* | -0·09*** | -0·19*** | 0·22*** | 0·01 | -0·07** | -0·06 | -0·10 |
|  | (-0·11, -0·08) | (-0·23, -0·16) | (0·18, 0·26) | (-0·03, 0·06) | (-0·09, -0·04) | (-0·11, -0·01) | (-0·16, -0·04) |
| *Cases×nurses* | -0·06** | -0·05 | 0·30*** | 0·03 | -0·02 | 0·05 | 0·02 |
|  | (-0·08, -0·04) | (-0·09, -0·02) | (0·23, 0·35) | (-0·01, 0·08) | (-0·04, 0·01) | 0·01, 0·10 | (-0·04, 0·08) |
| Panel B: Study II | | | | | | | |
| *Covid-19* | -0·02*** | 0·04*** | 0·15*** | -0·01 | 0·09*** | -0·03*** | 0·08*** |
|  | (-0·02, -0·01) | (0·03, 0·05) | (0·14, 0·15) | (-0·01, 0·00) | (0·08, 0·09) | (-0·04, -0·02) | (0·08, 0·08) |
| *Cases* | -0·02*** | 0·05*** | 0·17*** | 0·00 | 0·10*** | -0·04*** | 0·09*** |
|  | (-0·02, -0·01) | (0·04, 0·06) | (0·16, 0·17) | (-0·01, 0·01) | (0·09, 0·10) | (-0·05, -0·03) | (0·08, 0·09) |
| *Covid-19×cases* | -0·02*** | 0·05*** | 0·19*** | -0·01 | 0·11*** | -0·04*** | 0·10*** |
|  | (-0·02, -0·01) | (0·04, 0·07) | (0·19, 0·20) | (-0·02, 0·00) | (0·11, 0·12) | (-0·05, -0·03) | (0·10, 0·11) |
| Panel C: Study III | | | | | | | |
| *Covid-19* | -0·16** | -0·24** | 0·25 | -0·50* | -0·08 | -0·78* | -0·64** |
|  | (-0·17, -0·16) | (-0·25, -0·22) | (0·13, 0·37) | (-0·57, -0·44) | (-0·13, -0·02) | (-0·84, -0·72) | (-0·68, -0·61) |

Notes: 1. *=p<0·1, **=p<0·05, ***=p<0·01. 2. The brackets under coefficients are the 95% Confidence Interval. 3. K10 is an alternative measure of mental health. 4. Additional control of physical health and personalities was added. 5. Cases=the lawsuit cases of MV, Covid-19=the number of confirmed regional Covid-19 cases.

Table A6 Effects of ERI, job satisfaction, and organizational commitment on HP’s mental health

|  | Positive emotion | Negative emotion | Cognition | Social adaptability | Social contact | Social  support |
| --- | --- | --- | --- | --- | --- | --- |
|  | (1) | (2) | (3) | (4) | (5) | (6) |
| Panel A: Study I | | | | | | |
| ERI | -2·48*** | 1·22*** | -1·33*** | -1·08*** | -1·64*** | -1·52*** |
|  | (-2·62, -2·34) | (1·12, 1·33) | (-1·42, -1·25) | (-1·15, -1·00) | (-1·74, -1·54) | (-1·60, -1·43) |
| Observations | 5,304 | 5,304 | 5,304 | 5,304 | 5,304 | 5,304 |
| R-squared | 0·171 | 0·059 | 0·076 | 0·067 | 0·098 | 0·083 |
| Satisfaction | 0·50*** | -0·22*** | 0·31*** | 0·32*** | 0·35*** | 0·28*** |
|  | (0·48, 0·52) | (-0·25, -0·19) | (0·29, 0·33) | (0·29, 0·34) | (0·32, 0·37) | (0·26, 0·30) |
| Observations | 5,304 | 5,304 | 5,304 | 5,304 | 5,304 | 5,304 |
| R-squared | 0·186 | 0·056 | 0·094 | 0·118 | 0·113 | 0·080 |
| Organization support | 0·70*** | -0·07 | 0·54*** | 0·45*** | 0·59*** | 0·56*** |
|  | (0·67, 0·74) | (-0·13, -0·02) | (0·50, 0·58) | (0·40, 0·50) | (0·54, 0·64) | (0·50, 0·61) |
| Observations | 5,304 | 5,304 | 5,304 | 5,304 | 5,304 | 5,304 |
| R-squared | 0·095 | 0·033 | 0·069 | 0·063 | 0·075 | 0·067 |
| Panel B: Study II | | | | | | |
| ERI | -2·75*** | 2·10*** | -1·59*** | -1·36*** | -1·88*** | -1·58*** |
|  | (-3·04, -2·46) | (1·80, 2·39) | (-1·72, -1·45) | (-1·49, -1·22) | (-1·97, -1·79) | (-1·66, -1·49) |
| Observations | 5,589 | 5,589 | 5,589 | 5,589 | 5,589 | 5,589 |
| R-squared | 0·186 | 0·104 | 0·099 | 0·077 | 0·092 | 0·066 |
| Satisfaction | 0·71*** | -0·46*** | 0·41*** | 0·44*** | 0·48*** | 0·39*** |
|  | (0·68, 0·74) | (-0·50, -0·42) | (0·37, 0·45) | (0·43, 0·45) | (0·46, 0·50) | (0·37, 0·41) |
| Observations | 5,589 | 5,589 | 5,589 | 5,589 | 5,589 | 5,589 |
| R-squared | 0·237 | 0·101 | 0·120 | 0·142 | 0·116 | 0·076 |
| Organization support | 1·06*** | -0·24** | 0·71*** | 0·68*** | 0·87*** | 0·82*** |
|  | (0·98, 1·14) | (-0·35, -0·13) | (0·67, 0·75) | (0·65, 0·72) | (0·84, 0·90) | (0·77, 0·87) |
| Observations | 5,589 | 5,589 | 5,589 | 5,589 | 5,589 | 5,589 |
| R-squared | 0·116 | 0·025 | 0·085 | 0·075 | 0·078 | 0·067 |
| Panel C: Study III | | | | | | |
| *ERI* | -2·96** | 1·77 | -1·49** | -1·35* | -1·81** | -1·64** |
|  | (-3·08, -2·84) | (1·39, 2·14) | (-1·58, -1·40) | (-1·49, -1·20) | (-1·94, -1·69) | (-1·72, -1·55) |
| Observations | 6,886 | 6,886 | 6,886 | 6,886 | 6,886 | 6,886 |
| R-squared | 0·206 | 0·068 | 0·074 | 0·077 | 0·087 | 0·063 |
| *Satisfaction* | 0·59* | -0·31 | 0·35 | 0·36* | 0·40* | 0·37* |
|  | (0·50, 0·68) | (-0·42, -0·19) | (0·29, 0·41) | (0·32, 0·41) | (0·35, 0·45) | (0·34, 0·40) |
| Observations | 6,886 | 6,886 | 6,886 | 6,886 | 6,886 | 6,886 |
| R-squared | 0·195 | 0·050 | 0·091 | 0·124 | 0·100 | 0·076 |
| *Org_commitment* | 0·81 | -0·17 | 0·60 | 0·52 | 0·66 | 0·67 |
|  | (0·62, 1·01) | (-0·25, -0·09) | (0·49, 0·71) | (0·40, 0·64) | (0·54, 0·78) | (0·52, 0·82) |
| Observations | 6,886 | 6,886 | 6,886 | 6,886 | 6,886 | 6,886 |
| R-squared | 0·076 | 0·010 | 0·058 | 0·056 | 0·054 | 0·048 |

Notes: 1. *=p<0·1, **=p<0·05, ***=p<0·01. 2. The brackets under coefficients are the 95% Confidence Interval

Table A7 Moderate Effects of ERI, job satisfaction, and organizational commitment on HP’s mental health (Study I)

|  | Positive emotion | Negative emotion | Cognition | Social adaptability | Social contact | Social  support |
| --- | --- | --- | --- | --- | --- | --- |
|  | (1) | (2) | (3) | (4) | (5) | (6) |
| Panel A: ERI | | | | | | |
| *Cases×physicianv×ERI* | -0·86*** | 0·36*** | -0·44*** | -0·36*** | -0·57*** | -0·53*** |
|  | (-0·91,-0·81) | (0·32, 0·41) | (-0·47, -0·40) | (-0·39, -0·34) | (-0·61, -0·53) | (-0·56, -0·50) |
| *Cases×nurses×ERI* | -0·76*** | 0·42*** | -0·43*** | -0·34*** | -0·50*** | -0·46*** |
|  | (-0·80, -0·72) | (0·39, 0·46) | (-0·46, -0·40) | (-0·36, -0·31) | (-0·53, -0·47) | (-0·49, -0·42) |
| Observations | 5,304 | 5,304 | 5,304 | 5,304 | 5,304 | 5,304 |
| R-squared | 0·175 | 0·064 | 0·076 | 0·068 | 0·101 | 0·085 |
| Panel B: Job satisfaction | | | | | | |
| *Cases×physicianv×satisfaction* | 0·15*** | -0·08*** | 0·10*** | 0·10*** | 0·10*** | 0·08*** |
|  | (0·14, 0·16) | (-0·10, -0·07) | (0·09, 0·11) | (0·09, 0·11) | (0·10, 0·11) | (0·07, 0·09) |
| *Cases×nurses×satisfaction* | 0·17*** | -0·06*** | 0·10*** | 0·10*** | 0·12*** | 0·10*** |
|  | (0·16, 0·17) | (-0·07, -0·05) | (0·09, 0·11) | (0·10, 0·11) | (0·11, 0·12) | (0·09, 0·10) |
| Observations | 5,304 | 5,304 | 5,304 | 5,304 | 5,304 | 5,304 |
| R-squared | 0·188 | 0·063 | 0·094 | 0·118 | 0·114 | 0·081 |
| Panel C: Organization support | | | | | | |
| *Cases×physicianv×org_commitment* | 0·20*** | -0·04 | 0·18*** | 0·14*** | 0·17*** | 0·16*** |
|  | (0·18, 0·21) | (-0·06, -0·01) | (0·16, 0·19) | (0·12, 0·16) | (0·16, 0·19) | (0·14, 0·18) |
| *Cases×nurses×org_commitment* | 0·23*** | -0·02 | 0·18*** | 0·15*** | 0·20*** | 0·19*** |
|  | (0·22, 0·25) | (-0·03, 0·00) | (0·16, 0·19) | (0·13, 0·16) | (0·18, 0·21) | (0·17, 0·20) |
| Observations | 5,304 | 5,304 | 5,304 | 5,304 | 5,304 | 5,304 |
| R-squared | 0·099 | 0·040 | 0·069 | 0·064 | 0·077 | 0·069 |

Notes: 1.*=p<0·1, **=p<0·05, ***=p<0·01. 2. The brackets under coefficients are the 95% Confidence Interval.

Table A8 Moderate Effects of ERI, job satisfaction, and organizational commitment on HP’s mental health (Study II)

|  | Positive emotion | Negative emotion | Cognition | Social adaptability | Social contact | Social  support |
| --- | --- | --- | --- | --- | --- | --- |
|  | (1) | (2) | (3) | (4) | (5) | (6) |
| Panel A: ERI | | | | | | |
| *Covid-19×ERI* | -0·68*** | 0·53*** | -0·38*** | -0·34*** | -0·44*** | -0·37*** |
|  | (-0·72, -0·64) | (0·49, 0·57) | (-0·40, -0·35) | (-0·35, -0·32) | (-0·46, -0·41) | (-0·38, -0·36) |
| Observations | 3,883 | 3,883 | 3,883 | 3,883 | 3,883 | 3,883 |
| R-squared | 0·18 | 0·11 | 0·10 | 0·09 | 0·09 | 0·06 |
| *Cases×ERI* | -0·81*** | 0·63*** | -0·46*** | -0·40*** | -0·54*** | -0·45*** |
|  | (-0·90, -0·72) | (0·56, 0·70) | (-0·52, -0·39) | (-0·44, -0·36) | (-0·61, -0·46) | (-0·51, -0·39) |
| Observations | 5,589 | 5,589 | 5,589 | 5,589 | 5,589 | 5,589 |
| R-squared | 0·181 | 0·104 | 0·095 | 0·075 | 0·085 | 0·061 |
| *Cases×Covid-19×ERI* | -1·09*** | 0·86*** | -0·61*** | -0·54*** | -0·70*** | -0·59*** |
|  | (-1·17, -1·01) | (0·77, 0·94) | (-0·66, -0·55) | (-0·57, -0·50) | (-0·75, -0·65) | (-0·61, -0·56) |
| Observations | 3,851 | 3,851 | 3,851 | 3,851 | 3,851 | 3,851 |
| R-squared | 0·192 | 0·113 | 0·104 | 0·089 | 0·093 | 0·062 |
| Panel B: Job satisfaction | | | | | | |
| *Covid-19×satisfaction* | 0·17*** | -0·11*** | 0·10*** | 0·10*** | 0·11*** | 0·09*** |
|  | (0·15, 0·18) | (-0·12, -0·10) | (0·10, 0·11) | (0·09, 0·11) | (0·10, 0·12) | (0·09, 0·10) |
| Observations | 3,883 | 3,883 | 3,883 | 3,883 | 3,883 | 3,883 |
| R-squared | 0·225 | 0·105 | 0·134 | 0·141 | 0·112 | 0·075 |
| *Cases×satisfaction* | 0·20*** | -0·13*** | 0·12*** | 0·12*** | 0·13*** | 0·11*** |
|  | (0·17, 0·22) | (-0·14, -0·12) | (0·11, 0·12) | (0·10, 0·14) | (0·11, 0·15) | (0·10, 0·12) |
| Observations | 5,589 | 5,589 | 5,589 | 5,589 | 5,589 | 5,589 |
| R-squared | 0·220 | 0·099 | 0·116 | 0·129 | 0·105 | 0·071 |
| *Cases×Covid-19×satisfaction* | 0·27*** | -0·18*** | 0·16*** | 0·16*** | 0·17*** | 0·15*** |
|  | (0·24, 0·29) | (-0·20, -0·17) | (0·15, 0·17) | (0·14, 0·17) | (0·16, 0·19) | (0·14, 0·15) |
| Observations | 3,851 | 3,851 | 3,851 | 3,851 | 3,851 | 3,851 |
| R-squared | 0·238 | 0·111 | 0·137 | 0·149 | 0·118 | 0·078 |
| Panel C: Organization commitment | | | | | | |
| *Covid-19×org_commitment* | 0·26*** | -0·08*** | 0·17*** | 0·16*** | 0·19*** | 0·20*** |
|  | (0·24, 0·28) | (-0·09, -0·07) | (0·16, 0·18) | (0·15, 0·17) | (0·18, 0·21) | (0·19, 0·21) |
| Observations | 3,883 | 3,883 | 3,883 | 3,883 | 3,883 | 3,883 |
| R-squared | 0·117 | 0·026 | 0·091 | 0·082 | 0·076 | 0·068 |
| *Cases×org_commitment* | 0·30*** | -0·08*** | 0·20*** | 0·19*** | 0·24*** | 0·23*** |
|  | (0·27, 0·33) | (-0·09, -0·07) | (0·18, 0·22) | (0·17, 0·21) | (0·20, 0·28) | (0·21, 0·26) |
| Observations | 5,589 | 5,589 | 5,589 | 5,589 | 5,589 | 5,589 |
| R-squared | 0·110 | 0·026 | 0·080 | 0·070 | 0·070 | 0·065 |
| *Cases×Covid-19×org_commitment* | 0·41*** | -0·13*** | 0·27*** | 0·26** | 0·31*** | 0·31*** |
|  | (0·38, 0·44) | (-0·14, -0·11) | (0·25, 0·28) | (0·24, 0·28) | (0·28, 0·34) | (0·29, 0·33) |
| Observations | 3,851 | 3,851 | 3,851 | 3,851 | 3,851 | 3,851 |
| R-squared | 0·123 | 0·027 | 0·092 | 0·085 | 0·079 | 0·071 |

Notes: 1. *=p<0·1, **=p<0·05, ***=p<0·01. 2. The brackets under coefficients are the 95% Confidence Interval.

Table A9 Moderate Effects of ERI, job satisfaction, and organizational commitment on HP’s mental health (Study III)

|  | Positive emotion | Negative emotion | Cognition | Social adaptability | Social contact | Social  support |
| --- | --- | --- | --- | --- | --- | --- |
|  | (1) | (2) | (3) | (4) | (5) | (6) |
| Panel A: Treated, Covid-19 | | | | | | |
| *Covid-19×ERI* | -2·53 | 1·91 | -1·41 | -1·27 | -1·75 | -1·51 |
|  | (-3·44, -1·62) | (1·27, 2·55) | (-1·74, -1·08) | (-1·72, -0·83) | (-2·12, -1·38) | (-1·90, -1·13) |
| Observations | 6,886 | 6,886 | 6,886 | 6,886 | 6,886 | 6,886 |
| R-squared | 0·070 | 0·037 | 0·038 | 0·037 | 0·037 | 0·025 |
| *Covid-19×satisfactiom* | 0·61 | -0·40 | 0·36 | 0·37 | 0·38 | 0·33 |
|  | (0·40, 0·83) | (-0·56, -0·25) | (0·20, 0·52) | (0·24, 0·49) | (0·20, 0·56) | (0·17, 0·49) |
| Observations | 6,886 | 6,886 | 6,886 | 6,886 | 6,886 | 6,886 |
| R-squared | 0·076 | 0·033 | 0·042 | 0·050 | 0·033 | 0·022 |
| *Covid-19×org_commitment* | 0·69 | -0·14 | 0·43 | 0·46 | 0·45 | 0·51 |
|  | (0·11, 1·27) | (-0·36, 0·08) | (-0·03, 0·90) | (0·09, 0·82) | (-0·09, 1·00) | (-0·03, 1·05) |
| Observations | 6,886 | 6,886 | 6,886 | 6,886 | 6,886 | 6,886 |
| R-squared | 0·029 | 0·008 | 0·025 | 0·027 | 0·015 | 0·014 |
| Panel B: Treated, Covid-19+Cases | | | | | | |
| *Cases×Covid-19×ERI* | -0·66 | 0·50* | -0·36** | -0·33 | -0·45** | -0·39* |
|  | (-0·77, -0·55) | (0·42, 0·57) | (-0·39, -0·33) | (-0·39, -0·28) | (-0·47, -0·42) | (-0·42, -0·35) |
| Observations | 6,886 | 6,886 | 6,886 | 6,886 | 6,886 | 6,886 |
| R-squared | 0·076 | 0·040 | 0·039 | 0·039 | 0·039 | 0·026 |
| *Cases×Covid-19×satisfaction* | 0·16 | -0·10 | 0·09 | 0·09 | 0·10 | 0·08 |
|  | (0·13, 0·18) | (-0·13, -0·08) | (0·07, 0·11) | (0·08, 0·11) | (0·07, 0·12) | (0·06, 0·11) |
| Observations | 6,886 | 6,886 | 6,886 | 6,886 | 6,886 | 6,886 |
| R-squared | 0·083 | 0·036 | 0·046 | 0·054 | 0·038 | 0·025 |
| *Cases×Covid-19×org_commitment* | 0·20 | -0·04 | 0·13 | 0·13 | 0·14 | 0·15 |
|  | (0·10, 0·30) | (-0·09, 0·00) | (0·05, 0·21) | (0·07, 0·19) | (0·04, 0·24) | (0·06, 0·25) |
| Observations | 6,886 | 6,886 | 6,886 | 6,886 | 6,886 | 6,886 |
| R-squared | 0·035 | 0·008 | 0·029 | 0·031 | 0·019 | 0·018 |

Notes: 1. *=p<0·1, **=p<0·05, ***=p<0·01. 2. The brackets under coefficients are the 95% Confidence Interval.


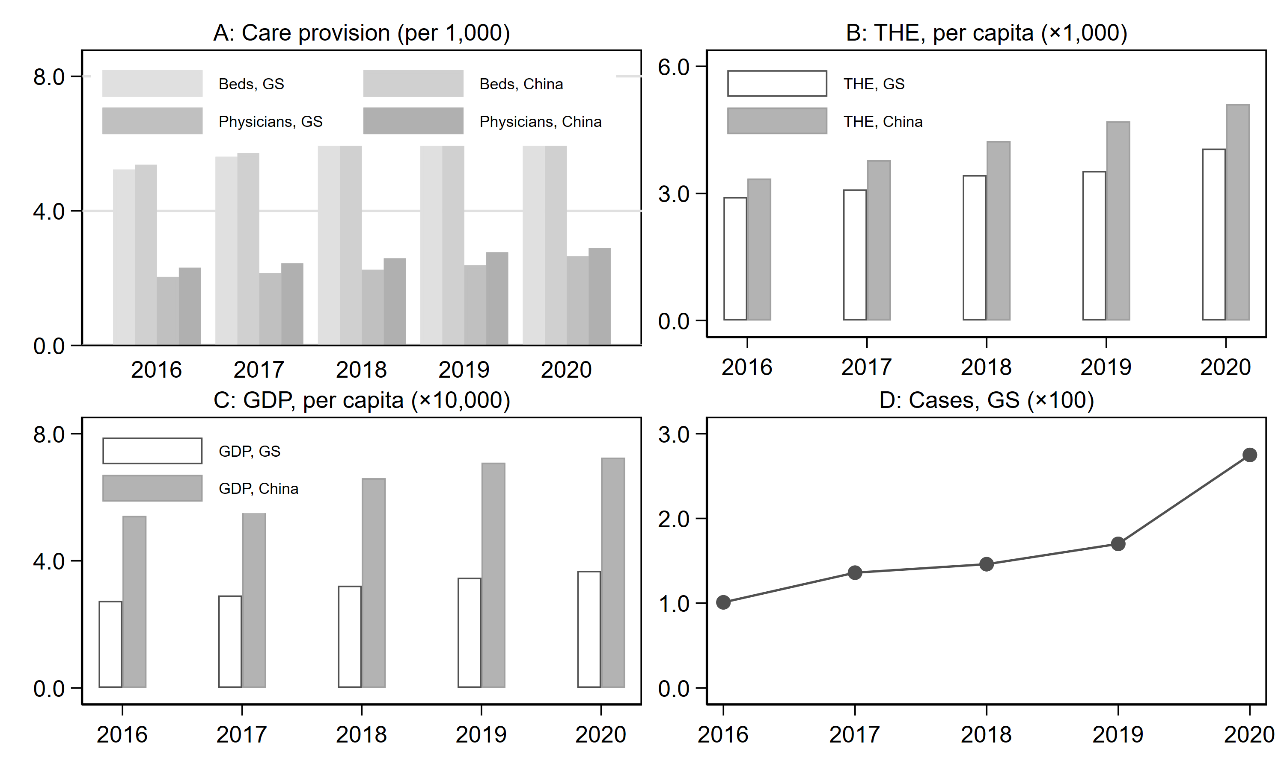


Figure A1 Healthcare provision, expenditure, GDP, and MV in GS, 2016-2020

Notes: This figure plots healthcare provision (denoted by beds and physicians per thousand), expenditure (per capita total health expenditure (THE)), economic development (per capita GDP), and the number of MV (indicated by the lawsuit cases related to the patient-physician relationship) in GS in the past 5 years (2016-2020). The unit of THE and GDP is CNY.

Figure A2 Survey design


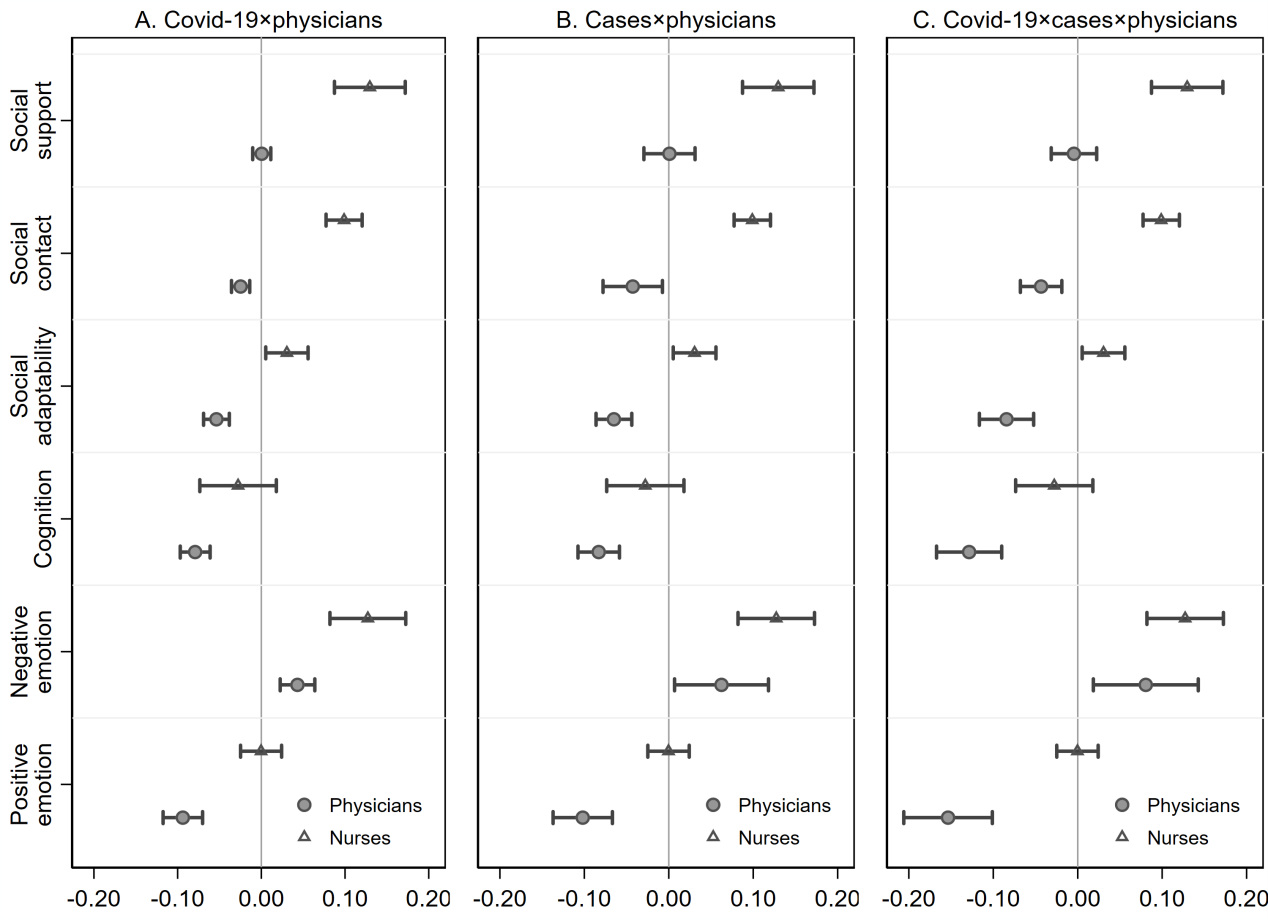


Figure A3 Impacts of MV, COVID-19, and their interaction on mental health of specific HP groups

Notes: The spots are coefficients, and the solid lines indicate 95% confidence intervals.
